# Dental Laws and Their Enforcement

**Published:** 1897-07

**Authors:** A. M. Callahan


ARTICLE X.

### DENTAL LAWS AND THEIR ENFORCEMENT.

BY DR. A. M. CALLAHAN.

Dental laws ars so universal that nothing on the subject is left for discussion except the provisions they should contain relative to their efficiency in securing the best services attainable for the people, and their justice to the dental profession.

The laws of Kansas recognize diplomas issued by reputable dental colleges, or certificates from our State Board of Dental Examiners. Some State laws require the applicant to be a graduate of some dental college, or hold a certificate from some other State board, to entitle him to examination, while other States require examination without any previous record of qualification.

There are some good points in a law requiring examination of all persons who apply for registration. It has a tendency to keep colleges and boards of examiners up to a high standard, and effectually kills fakir colleges and diploma mills.

In our own experience of six years as a member of a State board of dental examiners, we have found commendable improvement in the teaching of some dental colleges since the amendment to our law requiring examination of applicants who are not graduates.

While we believe a high standard should be maintained by State boards, we do not think it should be made so high as to be prohibitive.

At times I have thought our board was too lenient, and that too many passed and were granted certificates of registration. But a comparison of our examination papers with those of twenty other State boards shows but three others which require a more thorough knowledge of any branches, and they only in anatomy, histology, bacteriology and chemistry. One of these three State boards—Pennsylvania—we understand is prohibitive; no person having been able to pass their examination.

Only sixteen out of eighty examined in Illinois have passed.

We require eighty five per cent. in operative dentistry, and eighty per cent. in prosthetic, and a general average of seventy-five per cent. in anatomy, physiology, histology, pathology, materia medica, oral surgery, chemistry, metallurgy, crown- and bridge-work, and hygiene. The class of fourteen examined in April last made an excellent showing. Twelve of the fourteen passed. Eleven of the twelve made a general average above ninety per cent. In our class of 1896 we examined twenty-five, of whom twenty passed. Of this twenty, four had never attended dental college—one of the four stood at the head of the class. This is an instance where a law requiring the applicant to hold a diploma or a certificate from some other State board to entitle the applicant to the right of examination would have been an injustice.

In my judgment, our law should require every person who applies for registration to pass examination by the board. Those who are qualified need offer no objections to such a law, and those who are not should be debarred from practicing.

It should also make provision for a temporary license, or a license modeled after that granted to teachers—first, second and third grades; the last to be a grade that would entitle the holder to a certificate of registration, and the others limited as to time, and possibly as to the special work they should be permitted to do. This last cause is only offered as a suggestion for consideration. We have no doubt a law granting a temporary license would send many young men back to college to complete their dental education.

We are especially interested in the enforcement of the dental law. While it is probably as well enforced as the most of our laws, it could be made much more effective if the registered dentists would make a joint effort to enforce the law.

There seems to be a general impression that it is the duty of the board to prosecute violators of the law.

We frequently receive letters from dentists (some of them signed by the writer, and some anonymous) blowing up the law, and the board in general, and the Secretary in particular, for not having some violator punished.

These gentlemen are mistaken in the duties of the board. No such provision is made in the law. If it were so, then some method of raising a sufficient revenue to pay the prosecutor a salary and expenses would have been provided in the law.

The law puts it within the power of the registered to see it enforced. If it is inexpedient for any one dentist to prosecute a violator, on account of the prejudice of the people, you can do it jointly. Let the dentists of each judicial district form an organization, and elect an executive committee, whose duty it shall be, during the time they hold the office, to prosecute any violator who comes within their territory. To enable them to do so effectively, an amount of money should be raised sufficient to employ some active, pushing lawyer and pay him an agreed fee for every conviction he secures. When a violator comes within your district, let every dentist make an effort to secure the names of persons for whom he has done dental work, and the nature of that work, whether filling teeth or plate-work, and about the date when the work was done, and report the facts to the chairman of your executive committee. If each one tries to do his duty, you can soon secure sufficient evidence for your lawyer to make a sure conviction.

If this plan is adopted, I assure you the violators will give your district a wide berth—they will soon learn to "keep off the grass."

I know there are dentists who will lean back with their thumbs in the arm-holes of their vests and say, "Oh well, these petty violators don't disturb me. They never do any work for my patients. The poor devils need all they get. No, I won't join your association. This class of dentists forget they owe the community something. They owe something to the less prosperous dentist, who is striving to do his duty to his patients and the profession. They also owe something to the profession at large, whose standard is degraded by the violator. Of all who should take an active interest in the enforcement of the law, there is none other who should so energetically use his influence and his money in that direction as the well-to-do dentist.

As a general rule, when a violator comes into a neighborhood, some one writes the Secretary, who promptly notifies the violator, and he at once moves his camp. But this is not always so. The violator sometimes stays until he wears out in that community.

We sometimes succeed in getting the county attorney to prosecute such cases. The law makes it his duty, but he sometimes does it so reluctantly and in such a manner as to say that he hopes the jury will not find the defendant guilty. Hence the necessity of employing a lawyer.

There are a few registered dentists who assume the right to send their students or assistants to other towns and assume the responsibility of a guarantee that their work will be satisfactory. They think they are entitled to this privilege by virtue of their registration. This is a mistake. Our law is like the plan of salvation, in the fact that every one has to work out his own salvation.

Any person not registered, who is practicing dentistry in this State except as a student or assistant, and then only with his preceptor or employer, under his immediate instruction and direction, is held by the board as practicing in violation of law.

This ruling is approved by the Attorney-General.

In a few instances a registered dentist has taken into co-partnership with him a man who is not registered. I believe this registered dentist, as well as the one who sends his assistant out to violate the law, is himself violating the law in aiding another in the violation of the law, and some day soon I hope to be able to test that matter by prosecuting the registered dentist.

There are some noble exceptions to most of the people I have talked about, and of course, this association is formed of these exceptions.

The board extends its grateful appreciation to the dentists in this State and out of this State who have so faithfully given their best efforts to see the law enforced.

This paper is written with the hope that it may produce some discussion in the manner of the enforcement of the law, and that some better plan may be devised than has been suggested by the paper. The board is willing at all times to do all within its power to assist in the enforcement of the law, but there is no money in our treasury to pay the expenses of prosecutions.—*Western Dental Journal.*

